# Influence of surgical approach on heterotopic ossification after total hip arthroplasty – is minimal invasive better? A case control study

**DOI:** 10.1186/s12891-017-1391-x

**Published:** 2017-01-21

**Authors:** Maya Hürlimann, Filippo-Franco Schiapparelli, Niccolo Rotigliano, Enrique Testa, Felix Amsler, Michael T. Hirschmann

**Affiliations:** 1 Department of Orthopaedic Surgery and Traumatology, Kantonsspital Baselland (Bruderholz, Liestal, Laufen), CH-4101 Bruderholz, Switzerland; 2 Amsler Consulting, CH-4059 Basel, Switzerland; 3 University of Basel, Basel, Switzerland

**Keywords:** Heterotopic ossification, Anterolateral minimal invasive approach, Watson-Jones, Periarticular ossification, Total hip arthroplasty, THA, Transgluteal Bauer, Direct anterior approach

## Abstract

**Background:**

Heterotopic ossification (HO) is a well-known complication after total hip arthroplasty (THA).

Recently, the trend is to operate THA minimally invasive being less traumatic than standard approaches and promising a faster return to activity. The purpose of the study was to investigate if minimal invasive surgery (MIS), leads also to less HO after THA.

**Methods:**

This retrospective study included 134 consecutive patients undergoing THA. In 42 (31.3%) patients a standard modified anterolateral (STD-Watson-Jones), in 28 (20.9%) patients a standard transgluteal Bauer approach (STD-Bauer), in 39 (29.1%) a MIS direct anterior approach (AMIS) and in 25 (18.7%) patients a MIS anterolateral (MIS-AL) approach was used. Standard preoperative anterior-posterior and lateral radiographs were assessed for occurrence of HO. HO was classified according to Brooker. In addition, short- and long-term adverse events were noted. Data was statistically analyzed using Chi-square tests, analysis of variance, multivariate data analysis and Pearson’s correlation (*p* < 0.05).

**Results:**

Overall, HO was found in 38 caucasian patients (28.4%) after THA. The STD-Watson-Jones group showed the highest HO rate (45.2% *n* = 19) with a significant difference to the AMIS (23.1% *n* = 9) and STD-Bauer approach (14.3% *n* = 4). No statistical difference was found to the MIS-AL approach (24.0% *n* = 6). Postoperative complications did not differ significantly except for a higher incidence of Trendelenburg`s sign in STD-Bauer.

**Conclusions:**

The rate and degree of HO after THA were significantly different with regards to the surgical approach. The standard modified anterolateral approach resulted in the highest HO rate, however, MIS approaches showed higher HO rates than the STD-Bauer.

## Background

Heterotopic ossification (HO) is a well-known complication after total hip arthroplasty (THA) with a reported mean incidence of 24–32% [1–9]. There is only scarce evidence about the influence of the surgical approach on HO occurrence. Not more than two different approaches have yet been simultaneously compared in terms of HO [10, 11].

Although HO etiology remains unclear, it has been postulated that osteoinductive growth factors are released as consequence of soft tissue trauma inducing the formation of HO [12–14]. HO is believed to reach its complete formation after 6 to 12 weeks post-operative and not to progress anymore after this period [15]. Symptoms vary dependent on the severity of HO, and range from local pain to reduced range of hip motion [16, 17]. The Brooker’s classification grades HO into four different grades [18]. Most of the cases of HO belong to grade I and II and generally run asymptomatic being a collateral finding in routine follow-up radiographs [19]. A less incidence of patients present severe HO classified as grade III and IV, with more hip pain and significantly reduced ROM, flexion, abduction, and external rotation of the hip when compared to grades 0, I and II [15, 20].

In modern THA a considerable number of surgical approaches are used. Besides the conventional standard approaches, minimally invasive surgery (MIS) approaches are increasingly used. These MIS approaches promise less gluteal insufficiency, a faster rehabilitation and a quicker return to normal daily life activities [1, 21].

The primary purpose of the present study was to investigate the influence of the following surgical approaches on HO: minimally invasive anterolateral (MIS-AL), minimally invasive anterior (MIS-AMIS), standard transgluteal (STD-Bauer), and the standard modified anterolateral (STD-Watson-Jones). The secondary purpose was to investigate if minimally invasive surgery (MIS) leads to less HO than standard THA approaches.

## Methods

All patients who underwent primary THA in the period 2012–2013 in a university affiliated hospital with a minimum follow-up of 12 months and a signed informed consent were included in the present study. Patients without a preoperative and postoperative (at a least of 12 months) radiological control on radiographs and patients who did not sign the informed consent were not included. Indications for surgery were end-stage osteoarthritis and traumatic neck fractures. In this period the standard THA done was an uncemented Zweymüller type stem and a pressfit cup (Smith&Nephew, Switzerland).

In this period, four consultant surgeons of the same department performed a total of four different surgical approaches. Patients have been divided according to the performed surgical approach, standard modified anterolateral (STD-Watson-Jones) (group A), standard transgluteal Bauer approach (STD-Bauer) (group B), direct anterior minimally invasive (AMIS) (group C) and anterolateral minimally invasive (MIS-AL) (group D) (Table 1). The choice of the surgical approach was based on the preference of each surgeon.

**Table 1:** Patient demographics as well as HO and complications divided by the type of THA approach used

		Approach				Total
		Standard		Minimally invasive		
		STD Watson-Jones	STD Bauer	AMIS	MIS-AL	
Gender						
Men		21	16	22	10	69
Women		21	12	17	15	65
Mean age		42	28	39	25	
Comorbidities						
Previous THA		14	12	12	7	45
Diabetes mellitus		5	5	7	5	22
Cardiovascular diseases		24	15	22	15	76
Osteoporosis		5	1	1	3	10
HO						
Grade 0	Count	23	24	30	19	96
	%	19,0%	3,6%	7,7%	12,0%	
Grade 1	Count	8	1	3	3	15
	%	19,0%	3,6%	7,7%	12,0%	
Grade 2	Count	5	2	4	1	12
	%	11,9%	7,1%	10,3%	4,0%	
Grade 3	Count	3	0	2	1	6
	%	7,1%	0,0%	5,1%	4,0%	
Grade 4	Count	3	1	0	1	5
	%	7,1%	3,6%	0,0%	4,0%	
Total	Count	42	28	39	25	134
	%	31,3%	20,9%	29,1%	18,7%	
Mean Stay in hospital		42	28	39	25	
Intraoperative complications		4	3	1	0	8
Early complications						
Bleeding		1	2	0	0	3
Wound healing problems/infection		4	1	1	4	10
Cardiovascular events		1	1	4	0	6
Pulmonary embolism		0	0	1	0	1
Postoperative anemia		42	28	39	25	134
Urinary tract infection		5	1	0	0	6
Late complications						
Fracture		3	1	1	1	6
Dislocation		0	2	1	0	3
Loosening		1	0	0	0	1
Leg length discrepancy		9	9	14	7	39
Trendelenburg sign/muscular deficits		10	10	3	2	25
Local sensory disturbances/pain		7	5	9	6	27
Revision surgery		2	2	1	3	8

**Fig. 1:**
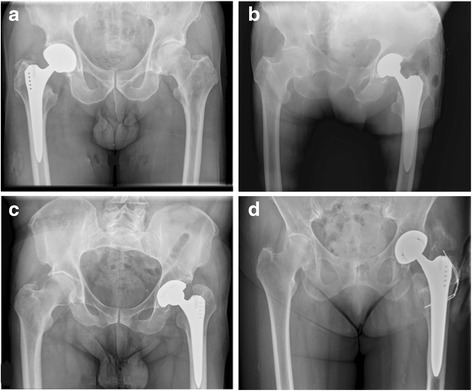
One year follow-up standard radiographic control, examples of heterotopic ossification according to Brooker grade 1 (**a**), grade 2 (**b**), grade 3 (**c**) and grade 4 (**d**)

In order to evaluate the HO onset and compare its incidence across the groups, pre-operative and 1-year post-operative radiological images (antero-posterior and lateral) were compared using a radiological display monitor (Fig. 1). The Brooker’s classification system was used (Table 1) [18].

Patients’ demographics including age, gender, relevant comorbidities (e.g. diabetes mellitus, cardiovascular diseases, osteoporosis, previous hip surgery) were noted. Intraoperative, early and late complications were obtained from the hospital archives system (KIS, Erne, Switzerland). The average time of hospital stay was calculated from hospital archives data.

Early mobilisation with full weight bearing was initiated under physiotherapeutic supervision on the first postoperative day. The postoperative protocol was identical for all groups.

### Statistical analysis

All data were analyzed by an independent statistician (F.A.) using SPSS version 17.0 (SPSS Inc., Chicago, IL, USA). Continuous variables were described using means and standard deviations or medians and ranges. Categorical variables were tabulated with absolute and relative frequencies. In the groups A-D no significant differences were seen in terms of gender, age and relevant comorbidities (*p* < 0.05). Univariate analysis was performed using Pearson´s correlation to identify any correlations between the type of approach, demographic and outcome variables. ANOVA analysis was also done for each variable. Multivariate analysis (MVA) was performed for HO incidence, occurred complications and time of hospital stay. To analyze a direct relationship between surgical approach and HO, all influencing additional factors were excluded (osteoporosis, fractures, dislocations, Trendelenburg`s sign/muscular deficit, muscular deficits, urinary tract infection) and a MVA was performed including all the results that showed univariate significance. Multivariate analysis used a stepwise linear regression of all variables on the dependent variable surgical approach. *p* < 0.05 was considered statistically significant and *p* < 0.1 as a statistical tendency.

## Results

One hundred thirty-four consecutive caucasian patients fulfilled the inclusion criteria. Forty-two patients (31.3%) were included in group A, 28 patients (20.9%) in group B, 39 patients (29.1%) in group C and 25 patients (18.7%) in group D (Table 1). HO was found in 28.4% of the patients with the highest incidence in group A (45.2%) followed by group D, (24.0%), group C (23.1%) and group B (14.3%) (Table 1). According to the Brooker’s classification, among the 28.4% patients who developed HO, 11.2% had a grade I, 9.0% a grade II, 4.5% a grade III and 3.7% a grade IV (Table 1) (Fig. 1a-d). Focusing on grade III and IV since they are the ones with clinical relevance: group A showed the highest HO rate, 14.2% (7.1% each for grade III and grade IV) followed by group D, 8.0% (4% each for grade III and grade IV); group C, 5.1% (5.1% grade III) and group B with an 3.6% incidence (3.6% grade IV) (Table 1). Group A developed significantly more HO than group B (*p* = 0.020) and group C (*p* = 0.038). Group A and group D showed no significant difference in terms of HO rate (*p* =0.095). Multivariate analysis showed that group A explained 5.0% of the factor "HO". In addition, the presence of osteoporosis explained 3.8% of the factor "HO". Overall, these two factors explained unadjusted 8.8% of the HO. Data about intraoperative, early and late complications are presented in Table 1. Urinary tract infection was significantly higher in group A (11.9%, *n* = 5) in comparison to group C (0.0%, *n* = 0; *p* = 0.022) and group D (0.0%, *n* = 0; *p* = 0.009). No statistical significant difference was seen in comparison to group B. (3.6%, *n* = 1, *p* = 0.095). The most frequent late complication was leg length discrepancy with an incidence of 29.1% in the overall study cohort. There was a significant difference between the groups (*p* < 0.01) for Trendelenburg`s sign indicating gluteal muscle insufficiency. It was seen in 35.7% of group B, 23.8% in of group A, 7.7% of group C and 8.0% of group D (Table 1). The average stay in hospital was 11.1 days (range 4–56 days) (Table 1). Patients in group C had a mean stay in hospital of 9.1 days, which was significantly shorter than group A (12.1 days), group B (11.5 days) and group D (12.1 days) (Table 1). The univariate Pearson’s correlation of all variables is shown in Table 2.

**Table 2:** Pearson correlation of demographic and outcome criteria (* = 0.05 ** = 0.01 *** = 0.001)

Pearson r	*N*	STD-Watson-Jones	STD-Bauer	AMIS	MIS-AL	MIS	Revision surgery	HO	Gender	Age	Previous THA
STD-Watson-Jones	134	1***	−0.35***	−0.43***	−0.32***	−0.65***	−0.03	0.22**	0.02	0.03	0
STD-Bauer	134	−0.35***	1***	−0.33***	−0.25**	−0.49***	0.03	−0.12	−0.06	0.01	0.1
AMIS	134	−0.43***	−0.33***	1***	−0.31***	0.67***	−0.09	−0.08	−0.06	0.07	−0.04
MIS-AL	134	−0.32***	−0.25**	−0.31***	1***	0.5***	0.12	−0.04	0.11	−0.12	−0.06
MIS	134	−0.65***	−0.49***	0.67***	0.5***	1***	0.01	−0.11	0.03	−0.03	−0.08
Revision surgery	134	−0.03	0.03	−0.09	0.12	0.01	1***	0.04	0.13	0.11	0.02
HO	134	0.22**	−0.12	−0.08	−0.04	−0.11	0.04	1***	0.11	0.14	0.16
Gender	134	0.02	−0.06	−0.06	0.11	0.03	0.13	0.11	1***	0.02	0.04
Age	134	0.03	0.01	0.07	−0.12	−0.03	0.11	0.14	0.02	1***	−0.07
Previous THA	134	0	0.1	−0.04	−0.06	−0.08	0.02	0.16	0.04	−0.07	1***
Diabetes	134	−0.08	0.02	0.03	0.05	0.06	−0.03	−0.05	0.09	0.09	−0.02
Cardiovascular diseases	134	0.01	−0.03	0	0.03	0.02	0.16	0.03	−0.24**	0.48***	−0.18*
Osteoporosis	134	0.11	−0.08	−0.12	0.08	−0.04	0.05	0.22*	0.24**	0.05	0.1
Hospital stay	134	0.13	0.04	−0.24**	0.09	−0.15	0.39***	0.17	0.2*	0.22*	0.12
Intraoperative complications	134	0.1	0.1	−0.09	−0.12	−0.18*	0.2*	0.07	0.13	0.16	0.02
Bleeding	134	0.01	0.17*	−0.1	−0.07	−0.14	−0.04	−0.08	0.05	0.03	0
Wound healing problems/ infections	134	0.05	−0.08	−0.12	0.16	0.01	0.41***	0.03	0.07	−0.06	−0.02
Cardiovascular events	134	−0.07	−0.02	0.18*	−0.1	0.08	0.1	−0.02	0.01	0.04	−0.15
Pulmonary embolism	134	−0.06	−0.04	0.14	−0.04	0.09	−0.02	−0.05	−0.08	0.05	0.12
Postoperative anemia	134	0.15	−0.05	−0.04	−0.07	−0.1	0.21*	0.06	0.17*	0.1	0
Urinary tract infections	134	0.24**	−0.02	−0.14	−0.1	−0.21*	0.1	0.15	0.15	0.07	0
Other neurological deficits/delirium	134	−0.01	0.13	−0.12	0.01	−0.1	−0.07	−0.07	−0.05	0.18*	0.04
Fractures	134	0.09	−0.02	−0.06	−0.01	−0.06	0.25**	0.19*	0.15	0.24**	0.08
Dislocation	134	−0.1	0.17*	0.01	−0.07	−0.04	0.17*	0.01	0.05	0.09	−0.11
Loosening	134	0.13	−0.04	−0.06	−0.04	−0.08	0.34***	0.12	−0.08	0	−0.06
Leg length discrepancy	134	−0.11	0.03	0.1	−0.01	0.08	−0.09	−0.1	0	−0.03	−0.04
Trendelenburg sign/muscular deficits	134	0.09	0.23**	−0.18*	−0.13	−0.27**	0.04	0.07	0.07	0.06	0.07
Local sensory disturbances/pain	134	−0.06	−0.03	0.05	0.05	0.08	0.19*	0.1	0.03	−0.12	0
Pearson r	*N*	Diabetes	Cardiovasc. diseases	Osteoporosis	Hospital stay	Intraoperative complications	Bleeding	Wound healing problems/infections	Cardiovascular events	Pulmonary embolism	Postoperative anemia
STD-Watson-Jones	134	−0.08	0.01	0.11	0.13	0.1	0.01	0.05	−0.07	−0.06	0.15
STD-Bauer	134	0.02	−0.03	−0.08	0.04	0.1	0.17*	−0.08	−0.02	−0.04	−0.05
AMIS	134	0.03	0	−0.12	−0.24**	−0.09	−0.1	−0.12	0.18*	0.14	−0.04
MIS-AL	134	0.05	0.03	0.08	0.09	−0.12	−0.07	0.16	−0.1	−0.04	−0.07
MIS	134	0.06	0.02	−0.04	−0.15	−0.18*	−0.14	0.01	0.08	0.09	−0.1
Revision surgery	134	−0.03	0.16	0.05	0.39***	0.2*	−0.04	0.41***	0.1	−0.02	0.21*
HO	134	−0.05	0.03	0.22*	0.17	0.07	−0.08	0.03	−0.02	−0.05	0.06
Gender	134	0.09	−0.24**	0.24**	0.2*	0.13	0.05	0.07	0.01	−0.08	0.17*
Age	134	0.09	0.48***	0.05	0.22*	0.16	0.03	−0.06	0.04	0.05	0.1
Previous THA	134	−0.02	−0.18*	0.1	0.12	0.02	0	−0.02	−0.15	0.12	0
Diabetes	134	1***	0.27**	0.03	−0.03	−0.03	−0.07	−0.05	0	0.2*	0.16
Cardiovasc. diseases	134	0.27**	1***	−0.15	0.1	0.09	0.03	0.08	−0.03	0.08	0.17*
Osteoporosis	134	0.03	−0.15	1***	0.12	0.05	−0.04	0.03	−0.06	−0.02	0.02
Hospital stay	134	−0.03	0.1	0.12	1***	0.19*	0.15	0.29***	−0.06	−0.07	0.12
Intraoperative complications	134	−0.03	0.09	0.05	0.19*	1***	0.39***	0.17	−0.05	−0.02	0.29***
Bleeding	134	−0.07	0.03	−0.04	0.15	0.39***	1***	−0.04	−0.03	−0.01	0.06
Wound healing problems/ infections	134	−0.05	0.08	0.03	0.29***	0.17	−0.04	1***	−0.06	−0.02	0.16
Cardiovasc. events	134	0	−0.03	−0.06	−0.06	−0.05	−0.03	−0.06	1***	−0.02	−0.1
Pulmonary embolism	134	0.2*	0.08	−0.02	−0.07	−0.02	−0.01	−0.02	−0.02	1***	0.19*
Postoperative anemia	134	0.16	0.17*	0.02	0.12	0.29***	0.06	0.16	−0.1	0.19*	1***
Urinary tract infections	134	0.1	0.04	0.08	0.12	0.1	−0.03	0.35***	−0.05	−0.02	0.18*
Other neurological deficits/delirium	134	−0.05	0.02	0.14	0.1	0.05	0.15	−0.08	−0.06	−0.02	0.09
Fractures	134	0.06	0.16	0.06	0.33***	0.58***	0.32***	0.52***	0.12	0.15	0.67***
Dislocation	134	0	0.12	0.08	0.21*	0.55***	−0.03	0.21*	−0.05	−0.02	0.28**
Loosening	134	−0.07	0.13	−0.04	−0.04	0.17*	0.32***	0.15	−0.03	−0.01	0.06
Leg length discrepancy	134	−0.04	0.08	−0.02	−0.02	−0.02	−0.01	−0.02	−0.02	−0.01	0.19*
Trendelenburg sign/muscular deficits	134	−0.02	−0.07	−0.12	−0.15	−0.02	0.01	0.07	0.18*	0.14	0.04
Local sensory disturbances/pain	134	−0.06	−0.01	0.16	0.07	0.04	0.06	0.01	−0.01	−0.04	0.08
Pearson r	N	Urinary tract infections	Other neurological Deficits/delirium	Fractures	Dislocation	Loosening	Leg length discrepancy	Trendelenburg sign/muscular deficits	Local sensory disturbances/pain		
STD-Watson-Jones	134	0.24**	−0.01	0.09	−0.1	0.13	−0.11	0.09	−0.06		
STD-Bauer	134	−0.02	0.13	−0.02	0.17*	−0.04	0.03	0.23**	−0.03		
AMIS	134	−0.14	−0.12	−0.06	0.01	−0.06	0.1	−0.18*	0.05		
MIS-AL	134	−0.1	0.01	−0.01	−0.07	−0.04	−0.01	−0.13	0.05		
MIS	134	−0.21*	−0.16	−0.06	−0.04	−0.08	0.08	−0.27**	0.08		
Revision surgery	134	0.1	0.36***	0.25**	0.17*	0.34***	−0.09	0.04	0.19*		
HO	134	0.15	0.04	0.19*	0.01	0.12	−0.1	0.07	0.1		
Gender	134	0.15	0.17*	0.15	0.05	−0.08	0	0.07	0.03		
Age	134	0.07	0.16	0.24**	0.09	0	−0.03	0.06	−0.12		
Previous THA	134	0	−0.01	0.08	−0.11	−0.06	−0.04	0.07	0		
Diabetes	134	0.1	0.06	0	−0.07	−0.04	−0.02	−0.06	−0.02		
Cardiovasc. diseases	134	0.04	0.16	0.12	0.13	0.08	−0.07	−0.01	−0.05		
Osteoporosis	134	0.08	0.06	0.08	−0.04	−0.02	−0.12	0.16	−0.07		
Hospital stay	134	0.12	0.33***	0.21*	−0.04	−0.02	−0.15	0.07	−0.09		
Intraoperative complications	134	0.1	0.58***	0.55***	0.17*	−0.02	−0.02	0.04	0.03		
Bleeding	134	−0.03	0.32***	−0.03	0.32***	−0.01	0.01	0.06	−0.08		
Wound healing problems/infections	134	0.35***	0.52***	0.21*	0.15	−0.02	0.07	0.01	0.14		
Cardiovascular events	134	−0.05	0.12	−0.05	−0.03	−0.02	0.18*	−0.01	0.07		
Pulmonary embolism	134	−0.02	0.15	−0.02	−0.01	−0.01	0.14	−0.04	−0.04		
Postoperative anemia	134	0.18*	0.67***	0.28**	0.06	0.19*	0.04	0.08	0.01		
Urinary tract infection	134	1***	0.5***	0.3***	−0.03	−0.02	0.1	0.08	0.16		
Other neurological deficits/delirium	134	0.21*	0.39***	−0.06	−0.04	−0.02	−0.06	0.01	−0.07		
Fractures	134	0.5***	1***	0.37***	0.2*	0.05	0.11	0.07	0.08		
Dislocation	134	0.3***	0.37***	1***	−0.03	−0.02	−0.06	0.17*	0.07		
Loosening	134	−0.03	0.2*	−0.03	1***	−0.01	0.13	0.06	0.18*		
Leg length discrepancy	134	−0.02	0.05	−0.02	−0.01	1***	−0.06	−0.04	0.17*		
Trendelenburg sign/muscular deficits	134	0.1	0.11	−0.06	0.13	−0.06	1***	0.03	0.05		
Local sensory disturbances/pain	134	0.08	0.07	0.17*	0.06	−0.04	0.03	1***	−0.05		

## Discussion

This is the first study comparing four different THA approaches in terms of HO. So far, only two types of surgical approaches were simultaneously compared. The overall incidence of HO in this study was 28.4%. Toom et al reported an HO incidence of 32% in 178 patients who underwent THA using a posterolateral approach [5]. Pavlou et al. noted an incidence of 24% in 39 patients who underwent THA using a STD-Watson-Jones approach [2]. Yanbin Zhu et al. reported a similar HO rate (30%) in a metanalysis involving 14 studies with a total of 6468 patients. However, the type of THA approach was not specified [4]. In summary, the overall HO incidence in the present study is in line with the previously reported HO rates in the published literature.

The most important finding of the present study was that the rate and degree of HO after THA was significantly dependent from the surgical approach used. The STD-Watson-Jones approach showed a significantly higher HO rate than the STD-Bauer and AMIS approaches. This was also higher than the MIS-AL approach but without any statistical significance. This last finding has been also noted by Repantis et al. [10]. In contrast to the present study Biz et al. found a higher HO rate for the STD-Bauer approach (*p* = 0.0163) when compared to the STD-Watson-Jones [11]. These different results could be related to the different type of used implants that included also patients who underwent a hemiprothesis. To date, there is not a single study comparing the HO rate of patients who underwent THA using the STD-Watson-Jones and AMIS approach. With regards to HO rates in patients after THA using the AMIS approach, the results are conflicting. Whereas Tippets et al. reported a HO rate of 41.5%, [22] which is higher than in the present study, Newman et al. reported a HO rate of 24.3% [6], which is comparable with this study. It could be speculated if the reason for the highest HO rate in the STD-Watson-Jones group lies in the more traumatic dissection, which is clearly less invasive using a MIS approach. A recent study of Unger et al. [23] found that the AMIS approach for THA comes along with less muscle damage and hematoma, shorter operative and exposure time, less bleeding and faster rehabilitation time. Although the highest HO rate was seen in the STD-Watson-Jones group, it was not possible to state that minimally invasive approaches lead to less HO. Indeed, both AMIS and MIS-AL had a higher HO incidence than the STD-Bauer. This finding remained unexplained.

In this study the lower complications’ rate with MIS (MIS-AL, AMIS) than with the standard techniques (STD-Bauer, STD-Watson-Jones) reflects the current knowledge and are considered as advantages of MIS as shown by Unger et al. [23]. However, among the complications, only the Trendelenburg sign was statistically significant for which the MVA showed an increased risk in the STD-Bauer group.

Another important finding of this study was the direct comparison of the stay in hospital among the four approaches. Patients who underwent THA using an AMIS approach had the shortest mean hospital stay. However, the average stay in hospital of the MIS-AL group was probably distorted by a patient, who developed an early infection, has been three times operated and remained in the hospital 56.0 days. The stay in hospital difference between AMIS and the STD-Watson-Jones was the only statistically significant difference.

So far, no study compared four surgical approaches for a THA with regards to the hospital stay. Ilchmann et. al. [24] noted a significantly shorter stay in hospital after the AMIS approach compared to the STD-Bauer approach. Yue et. al. found similar results in a meta-analysis [25]. However, the average stay in hospital was 9.1 days for the AMIS and 11.5 days for the STD-Bauer approach (Table 1). All together MIS approaches for THA appear to be beneficial in terms of HO as well as adverse events. Hence, if possible MIS approaches should be used. In conclusion, this study has proven the general superiority of MIS approaches in terms of HO and adverse events.

A considerable number of limitations need to be considered. The most important one is the retrospective study design. The possible selection bias was however limited by the consecutive patient selection.

Four different orthopedic surgeon operated on the patients reported and choose the approach with regards to their own preference, which might have influenced the outcome in each group. However, all surgeons were capable to perform all four different approaches. As it was the aim to focus on HO after THA no clinical outcome data was assessed. Furthermore, only patients with HO grades 3 and 4 were included. Patients, who developed complications during couse of treatment were also included, which could have led to a longer stay in hospital as well as increased HO development.

The major strength of the study presented is that it is the first study investigating HO onset in four different THA approaches. In addition, it represents a consecutive patient series with a good and balanced sample size.

## Conclusion

This is the first study comparing the STD-Watson-Jones, STD-Bauer, AMIS and MIS-AL approaches in terms of HO. The rate and degree of HO after THA was significantly dependent from the surgical approach used. The STD-Watson-Jones presented the highest HO rate. A lower complications’ rate was seen after minimally invasive approaches. Hence, if possible MIS approaches should be used. In conclusion, this study has proven the general superiority of MIS approaches in terms of HO and adverse events.

## Abbreviations

AMIS: Minimally invasive direct anterior approach
HO: Heterotopic ossifications
MIS: Minimal invasive surgery
MIS-AL: Minimally invasive anterolateral
MVA: Multivariate analysis
STD-Bauer: Standard transgluteal Bauer approach
STD-Watson-Jones: Standard modified anterolateral Watson-Jones approach
THA: Total hip arthroplasty
